# Poly[[aqua­calcium(II)]-μ_4_-1*H*-imidazole-4,5-dicarboxyl­ato]

**DOI:** 10.1107/S1600536810039000

**Published:** 2010-10-09

**Authors:** Ya-Ting Chang, Chun-Ting Yeh, Ching-Che Kao, Chia-Her Lin

**Affiliations:** a Department of Chemistry, Chung-Yuan Christian University, Chung-Li 320, Taiwan

## Abstract

In the title compound, [Ca(C_5_H_2_N_2_O_4_)(H_2_O)]_*n*_, the Ca^2+^ cations are eigthtfold coordinated by six O atoms and one N atom of four symmetry-related anions and one water mol­ecule within an irregular polyhedron. These CaO_7_N polyhedra are connected *via* the anions into a three-dimensional network. The anions are additionally linked by N—H⋯O and O—H⋯O hydrogen bonding.

## Related literature

For general background to metal coordination polymers, see: Kitagawa *et al.* (2004 ▶). For related structures, see: Gao *et al.* (2004 ▶); Starosta & Leciejewicz (2006 ▶). 
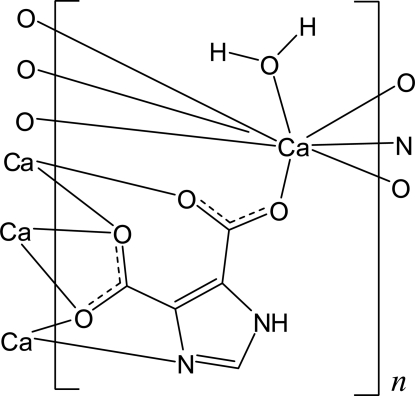


## Experimental

### 

#### Crystal data

[Ca(C_5_H_2_N_2_O_4_)(H_2_O)]
*M* *_r_* = 212.18
Monoclinic, 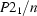

*a* = 6.4752 (4) Å
*b* = 9.7627 (6) Å
*c* = 10.9079 (6) Å
β = 103.041 (2)°
*V* = 671.76 (7) Å^3^
*Z* = 4
Mo *K*α radiation
μ = 0.92 mm^−1^
*T* = 295 K
0.45 × 0.20 × 0.15 mm

#### Data collection

Bruker APEXII CCD diffractometer
Absorption correction: multi-scan (*SADABS*; Bruker, 2008) ▶ *T*_min_ = 0.681, *T*_max_ = 0.874
6029 measured reflections
1665 independent reflections
1619 reflections with *I* > 2σ(*I*)
*R*_int_ = 0.024

#### Refinement

*R*[*F* ^2^ > 2σ(*F* ^2^)] = 0.025
*wR*(*F* ^2^) = 0.069
*S* = 1.08
1665 reflections
119 parameters
H-atom parameters constrained
Δρ_max_ = 0.33 e Å^−3^
Δρ_min_ = −0.35 e Å^−3^

### 

Data collection: *APEX2* (Bruker, 2010 ▶); cell refinement: *SAINT* (Bruker, 2010 ▶); data reduction: *SAINT*; program(s) used to solve structure: *SHELXS97* (Sheldrick, 2008 ▶); program(s) used to refine structure: *SHELXL97* (Sheldrick, 2008 ▶); molecular graphics: *SHELXTL* (Sheldrick, 2008 ▶); software used to prepare material for publication: *SHELXTL*.

## Supplementary Material

Crystal structure: contains datablocks I, global. DOI: 10.1107/S1600536810039000/nc2199sup1.cif

Structure factors: contains datablocks I. DOI: 10.1107/S1600536810039000/nc2199Isup2.hkl

Additional supplementary materials:  crystallographic information; 3D view; checkCIF report

## Figures and Tables

**Table 1:** Hydrogen-bond geometry (Å, °)

*D*—H⋯*A*	*D*—H	H⋯*A*	*D*⋯*A*	*D*—H⋯*A*
O5—H5*A*⋯O4^i^	0.85	2.13	2.9552 (14)	162
O5—H5*B*⋯O1^ii^	0.85	2.23	3.0109 (14)	153
N2—H2*A*⋯O4^iii^	0.86	1.86	2.7220 (13)	176

Symmetry codes: (i) 
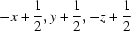
; (ii) 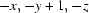; (iii) 
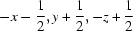
.

**Fig. 1.**
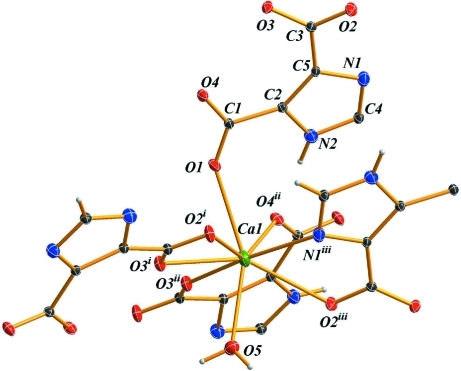
Crystal structure of the title compound with labelling and displacement ellipsoids drawn at the 50% probability level. Symmetry codes: (i) x + 1/2, -y + 1/2, z - 1/2; (ii) -x - 1/2, y + 1/2, -z + 1/2; (iii) -x, -y + 1, -z + 1.

**Fig. 2.**
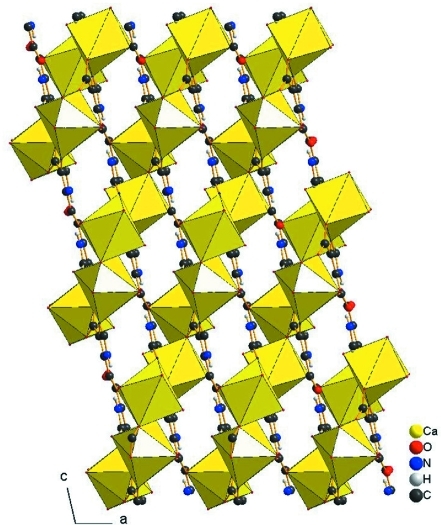
Crystal structure of the title compound with view along the crystallographic b axis.

## supplementary crystallographic information

### Comment

The synthesis of metal coordination polymers has been a intense research due to their interesting topologies and potential applications (Kitagawa, *et al.*, 2004). The imidazole-4,5-dicarboxylic acid (H_3_IDC) has been successively applied to construct two calcium complexes (Gao, *et al.*, 2004; Starosta, *et al.*, 2006). In our ongoing investigations in this field we report here the structure of a new Ca compound with the anionic imidazole-4,5-dicarboxylato ligand.

The asymmetric unit of the title compound conists of one Ca atom, one carboxylate ligand and one coordinated water molecule all of them located in general positions (Figure 1). The Ca center is eight-coordinated by six oxygen atoms and one nitrogen atom of four carboxylate ligands and one oxygen atom of a coordinated water molecule within an irregular polyhedron. The Ca—O distances range from 2.3197 (11) to 2.8777 (12) Å and the Ca—N distance amount to 2.4215 (10)Å. The CaO_7_N polyhedra are connected via the anions into a three-dimensional network and are further linked by N—H···O and O—H···O hydrogen bonding (Fig. 2 and Table 1).

### Experimental

imidazole-4,5-dicarboxylic acid (C_5_H_4_N_2_O_4_, 0.0752 g, 0.45 mmol) and Ca(NO_3_)_2_.4H_2_O (0.2361 g, 1 mmol) were reacted in 10 mL of H_2_O in a Teflon-lined digestion bomb with an internal volume of 23 ml l. The reaction mixture was heated to 453 K for 5 d followed by slow cooling at 6 K/h to room temperature. The product consits of transparent colorless crystals.

### Refinement

H atoms were constrained to ideal geometries, with C—H = 0.93 Å, O—H = 0.85 Å and N—H = 0.86 Å and refined with *U*_iso_(H) = 1.2*U*_eq_(1.5 for water H atoms) using a riding model.

### Crystal data

**Table:**

[Ca(C_5_H_2_N_2_O_4_)(H_2_O)]	*F*(000) = 432
*M**_r_* = 212.18	*D*_x_ = 2.098 Mg m^−^^3^
Monoclinic, *P*2_1_/*n*	Mo *K*α radiation, λ = 0.71073 Å
Hall symbol: -P 2yn	Cell parameters from 4091 reflections
*a* = 6.4752 (4) Å	θ = 2.8–28.3°
*b* = 9.7627 (6) Å	µ = 0.92 mm^−^^1^
*c* = 10.9079 (6) Å	*T* = 295 K
β = 103.041 (2)°	Columnar, colourless
*V* = 671.76 (7) Å^3^	0.45 × 0.20 × 0.15 mm
*Z* = 4	

### Data collection

**Table:**

Bruker APEXII CCD diffractometer	1665 independent reflections
Radiation source: fine-focus sealed tube	1619 reflections with *I* > 2σ(*I*)
graphite	*R*_int_ = 0.024
Detector resolution: 8.3333 pixels mm^-1^	θ_max_ = 28.3°, θ_min_ = 2.8°
φ and ω scans	*h* = −8→8
Absorption correction: multi-scan (*SADABS*; Bruker, 2008)	*k* = −12→12
*T*_min_ = 0.681, *T*_max_ = 0.874	*l* = −14→14
6029 measured reflections	

### Refinement

**Table:**

Refinement on *F*^2^	Secondary atom site location: difference Fourier map
Least-squares matrix: full	Hydrogen site location: inferred from neighbouring sites
*R*[*F*^2^ > 2σ(*F*^2^)] = 0.025	H-atom parameters constrained
*wR*(*F*^2^) = 0.069	*w* = 1/[σ^2^(*F*_o_^2^) + (0.0397*P*)^2^ + 0.2825*P*] where *P* = (*F*_o_^2^ + 2*F*_c_^2^)/3
*S* = 1.08	(Δ/σ)_max_ < 0.001
1665 reflections	Δρ_max_ = 0.33 e Å^−^^3^
119 parameters	Δρ_min_ = −0.35 e Å^−^^3^
0 restraints	Extinction correction: *SHELXL97* (Sheldrick, 2008), Fc^*^=kFc[1+0.001xFc^2^λ^3^/sin(2θ)]^-1/4^
Primary atom site location: structure-invariant direct methods	Extinction coefficient: 0.092 (5)

### Special details

**Table:**

Geometry. All esds (except the esd in the dihedral angle between two l.s. planes) are estimated using the full covariance matrix. The cell esds are taken into account individually in the estimation of esds in distances, angles and torsion angles; correlations between esds in cell parameters are only used when they are defined by crystal symmetry. An approximate (isotropic) treatment of cell esds is used for estimating esds involving l.s. planes.
Refinement. Refinement of *F*^2^ against ALL reflections. The weighted *R*-factor *wR* and goodness of fit *S* are based on *F*^2^, conventional *R*-factors *R* are based on *F*, with *F* set to zero for negative *F*^2^. The threshold expression of *F*^2^ > σ(*F*^2^) is used only for calculating *R*-factors(gt) *etc*. and is not relevant to the choice of reflections for refinement. *R*-factors based on *F*^2^ are statistically about twice as large as those based on *F*, and *R*- factors based on ALL data will be even larger.

### Fractional atomic coordinates and isotropic or equivalent isotropic displacement parameters (Å^2^)

**Table:**

	*x*	*y*	*z*	*U*_iso_*/*U*_eq_	
Ca1	0.08356 (4)	0.55299 (2)	0.17359 (2)	0.01629 (12)	
O1	−0.22252 (16)	0.39733 (10)	0.20098 (8)	0.0226 (2)	
O2	−0.25830 (16)	0.22314 (10)	0.70724 (8)	0.0239 (2)	
O3	−0.40728 (15)	0.13090 (9)	0.52331 (8)	0.0202 (2)	
O4	−0.30507 (15)	0.19962 (9)	0.28239 (8)	0.0201 (2)	
O5	0.38501 (16)	0.60082 (11)	0.08033 (10)	0.0293 (2)	
H5A	0.5147	0.6114	0.1164	0.044*	
H5B	0.3763	0.5845	0.0028	0.044*	
C1	−0.26916 (17)	0.32726 (12)	0.28690 (10)	0.0140 (2)	
C2	−0.27309 (17)	0.39902 (11)	0.40685 (10)	0.0132 (2)	
C3	−0.31453 (17)	0.22884 (12)	0.58996 (11)	0.0143 (2)	
C4	−0.23120 (19)	0.57835 (12)	0.53133 (11)	0.0160 (2)	
H4A	−0.2128	0.6687	0.5586	0.019*	
C5	−0.27759 (17)	0.35991 (11)	0.52820 (10)	0.0129 (2)	
N1	−0.24822 (16)	0.47378 (10)	0.60526 (9)	0.0152 (2)	
N2	−0.24362 (16)	0.53860 (10)	0.41286 (10)	0.0147 (2)	
H2A	−0.2347	0.5911	0.3510	0.018*	

### Atomic displacement parameters (Å^2^)

**Table:**

	*U*^11^	*U*^22^	*U*^33^	*U*^12^	*U*^13^	*U*^23^
Ca1	0.02062 (16)	0.01716 (16)	0.01057 (16)	0.00256 (8)	0.00247 (10)	−0.00080 (8)
O1	0.0348 (5)	0.0199 (5)	0.0159 (4)	−0.0034 (4)	0.0118 (4)	0.0003 (3)
O2	0.0332 (5)	0.0246 (5)	0.0121 (4)	−0.0058 (4)	0.0009 (4)	0.0035 (3)
O3	0.0297 (5)	0.0141 (4)	0.0160 (4)	−0.0067 (3)	0.0032 (4)	−0.0002 (3)
O4	0.0305 (5)	0.0138 (4)	0.0184 (4)	−0.0033 (3)	0.0103 (4)	−0.0037 (3)
O5	0.0252 (5)	0.0310 (6)	0.0326 (5)	−0.0034 (4)	0.0082 (4)	−0.0045 (4)
C1	0.0155 (5)	0.0141 (5)	0.0128 (5)	0.0003 (4)	0.0038 (4)	−0.0012 (4)
C2	0.0151 (5)	0.0111 (5)	0.0133 (5)	−0.0002 (4)	0.0032 (4)	−0.0001 (4)
C3	0.0159 (5)	0.0141 (5)	0.0130 (5)	−0.0004 (4)	0.0033 (4)	0.0015 (4)
C4	0.0197 (5)	0.0125 (5)	0.0157 (5)	−0.0007 (4)	0.0041 (4)	−0.0023 (4)
C5	0.0146 (5)	0.0120 (5)	0.0116 (5)	−0.0011 (4)	0.0020 (4)	−0.0014 (4)
N1	0.0194 (5)	0.0131 (4)	0.0129 (5)	−0.0015 (4)	0.0030 (4)	−0.0026 (4)
N2	0.0196 (5)	0.0115 (5)	0.0138 (5)	−0.0003 (3)	0.0054 (4)	0.0009 (3)

### Geometric parameters (Å, °)

**Table:**

Ca1—O3^i^	2.3211 (9)	O3—Ca1^v^	2.4408 (9)
Ca1—N1^ii^	2.4215 (10)	O4—C1	1.2665 (14)
Ca1—O4^i^	2.4336 (9)	O4—Ca1^vi^	2.4336 (9)
Ca1—O3^iii^	2.4408 (9)	O5—H5A	0.8497
Ca1—O5	2.4411 (11)	O5—H5B	0.8496
Ca1—O1	2.5679 (10)	C1—C2	1.4895 (15)
Ca1—O2^ii^	2.6614 (10)	C2—N2	1.3755 (14)
Ca1—O2^iii^	2.8776 (10)	C2—C5	1.3842 (15)
Ca1—C3^iii^	3.0181 (12)	C3—C5	1.4904 (16)
Ca1—Ca1^iv^	3.8384 (5)	C3—Ca1^v^	3.0182 (12)
O1—C1	1.2511 (14)	C4—N1	1.3209 (16)
O2—C3	1.2498 (14)	C4—N2	1.3342 (15)
O2—Ca1^ii^	2.6613 (10)	C4—H4A	0.9300
O2—Ca1^v^	2.8777 (10)	C5—N1	1.3806 (14)
O3—C3	1.2675 (14)	N1—Ca1^ii^	2.4215 (10)
O3—Ca1^vi^	2.3212 (9)	N2—H2A	0.8600
			
O3^i^—Ca1—N1^ii^	166.21 (3)	O5—Ca1—Ca1^iv^	73.40 (3)
O3^i^—Ca1—O4^i^	76.01 (3)	O1—Ca1—Ca1^iv^	84.38 (2)
N1^ii^—Ca1—O4^i^	92.65 (3)	O2^ii^—Ca1—Ca1^iv^	134.22 (2)
O3^i^—Ca1—O3^iii^	72.60 (3)	O2^iii^—Ca1—Ca1^iv^	83.473 (19)
N1^ii^—Ca1—O3^iii^	121.18 (3)	C3^iii^—Ca1—Ca1^iv^	59.22 (2)
O4^i^—Ca1—O3^iii^	134.15 (3)	C1—O1—Ca1	137.21 (8)
O3^i^—Ca1—O5	79.88 (4)	C3—O2—Ca1^ii^	117.19 (8)
N1^ii^—Ca1—O5	102.83 (4)	C3—O2—Ca1^v^	84.15 (7)
O4^i^—Ca1—O5	131.87 (3)	Ca1^ii^—O2—Ca1^v^	158.66 (4)
O3^iii^—Ca1—O5	73.62 (3)	C3—O3—Ca1^vi^	148.04 (8)
O3^i^—Ca1—O1	94.03 (3)	C3—O3—Ca1^v^	104.46 (7)
N1^ii^—Ca1—O1	89.89 (3)	Ca1^vi^—O3—Ca1^v^	107.40 (3)
O4^i^—Ca1—O1	72.51 (3)	C1—O4—Ca1^vi^	135.14 (8)
O3^iii^—Ca1—O1	77.25 (3)	Ca1—O5—H5A	129.0
O5—Ca1—O1	150.74 (3)	Ca1—O5—H5B	119.9
O3^i^—Ca1—O2^ii^	104.41 (3)	H5A—O5—H5B	108.7
N1^ii^—Ca1—O2^ii^	63.83 (3)	O1—C1—O4	125.60 (10)
O4^i^—Ca1—O2^ii^	70.90 (3)	O1—C1—C2	117.11 (10)
O3^iii^—Ca1—O2^ii^	149.31 (3)	O4—C1—C2	117.22 (10)
O5—Ca1—O2^ii^	75.80 (3)	N2—C2—C5	105.09 (10)
O1—Ca1—O2^ii^	133.11 (3)	N2—C2—C1	118.56 (10)
O3^i^—Ca1—O2^iii^	120.77 (3)	C5—C2—C1	135.93 (10)
N1^ii^—Ca1—O2^iii^	73.01 (3)	O2—C3—O3	122.99 (11)
O4^i^—Ca1—O2^iii^	141.64 (3)	O2—C3—C5	117.48 (10)
O3^iii^—Ca1—O2^iii^	48.30 (3)	O3—C3—C5	119.48 (10)
O5—Ca1—O2^iii^	86.41 (3)	O2—C3—Ca1^v^	71.53 (7)
O1—Ca1—O2^iii^	72.09 (3)	O3—C3—Ca1^v^	51.54 (6)
O2^ii^—Ca1—O2^iii^	127.387 (11)	C5—C3—Ca1^v^	170.99 (8)
O3^i^—Ca1—C3^iii^	96.57 (3)	N1—C4—N2	111.80 (11)
N1^ii^—Ca1—C3^iii^	97.22 (3)	N1—C4—H4A	124.1
O4^i^—Ca1—C3^iii^	144.90 (3)	N2—C4—H4A	124.1
O3^iii^—Ca1—C3^iii^	23.99 (3)	N1—C5—C2	109.29 (10)
O5—Ca1—C3^iii^	78.38 (3)	N1—C5—C3	115.48 (10)
O1—Ca1—C3^iii^	73.91 (3)	C2—C5—C3	135.17 (10)
O2^ii^—Ca1—C3^iii^	142.98 (3)	C4—N1—C5	105.64 (10)
O2^iii^—Ca1—C3^iii^	24.33 (3)	C4—N1—Ca1^ii^	127.80 (8)
O3^i^—Ca1—Ca1^iv^	37.36 (2)	C5—N1—Ca1^ii^	119.26 (7)
N1^ii^—Ca1—Ca1^iv^	156.42 (3)	C4—N2—C2	108.16 (10)
O4^i^—Ca1—Ca1^iv^	107.26 (2)	C4—N2—H2A	125.9
O3^iii^—Ca1—Ca1^iv^	35.24 (2)	C2—N2—H2A	125.9

Symmetry codes: (i) −*x*−1/2, *y*+1/2, −*z*+1/2; (ii) −*x*, −*y*+1, −*z*+1; (iii) *x*+1/2, −*y*+1/2, *z*−1/2; (iv) −*x*, −*y*+1, −*z*; (v) *x*−1/2, −*y*+1/2, *z*+1/2; (vi) −*x*−1/2, *y*−1/2, −*z*+1/2.

### Hydrogen-bond geometry (Å, °)

**Table:**

*D*—H···*A*	*D*—H	H···*A*	*D*···*A*	*D*—H···*A*
O5—H5A···O4^vii^	0.85	2.13	2.9552 (14)	162
O5—H5B···O1^iv^	0.85	2.23	3.0109 (14)	153
N2—H2A···O4^i^	0.86	1.86	2.7220 (13)	176

Symmetry codes: (vii) −*x*+1/2, *y*+1/2, −*z*+1/2; (iv) −*x*, −*y*+1, −*z*; (i) −*x*−1/2, *y*+1/2, −*z*+1/2.
